# Franklin County (Pennsylvania) Veterinary Society

**Published:** 1890-09

**Authors:** 


*Franklin County* (*Penna.*) *Veterinary Society.*—The veterinary surgeons of Franklin County, Pa., who are graduates of veterinary colleges, met in the council hall at Chambersburg, on Wednesday, the 29th inst., and organized a county society. There were present Drs. Smith, of Chambersburg; Kuhn and Bradley, of Mercersburg; Brindle, of Scotland; Snively, of Greencastle, and Brake, of St. Thomas. Dr. Smith was elected president, Dr. Kuhn, secretary, and Dr. Brake, treasurer. A committee was appointed to draft a constitution and by-laws to report at a meeting to be held September 24th.

